# The Effect of Water during the Compaction Process on Surface Characteristics of HMA Pavement

**DOI:** 10.3390/ma17092146

**Published:** 2024-05-03

**Authors:** Bingquan Dai, Lei Mao, Pan Pan, Xiaodi Hu, Ning Wang

**Affiliations:** 1 School of Civil Engineering and Architecture, Wuhan Institute of Technology, Wuhan 430205, China; 2 Hubei Provincial Engineering Research Center for Green Civil Engineering Materials and Structures, Wuhan 430073, China; 3 Hubei Communication Investment Intelligent Detection Co., Ltd., Wuhan 430100, China; 4 China Three Gorges Construction Engineering Corporation, Chengdu 610095, China

**Keywords:** HMA pavement, water consumption, surface polishing, black pixel ratio, mass loss ratio, molding temperature

## Abstract

During the compaction process of HMA pavement, it is common to spray cold water on the wheel of a road roller to prevent the mixture from sticking to the wheel, which might deteriorate the bonding strength between the asphalt binder and aggregate, and consequently lead to surface polishing of the pavement. This paper aims to demonstrate whether the water used during the compaction process affects the surface performance of HMA pavement. In this study, the black pixel ratio and mass loss ratio were used to evaluate the water effect on the surface performance of asphalt pavement, considering the water consumption, molding temperature and long-term ageing process. The test results indicated that the water used during the compaction process would increase the risk of surface polishing of HMA pavement. This adverse effect became more significant if the HMA samples were prepared using greater water consumption, a greater molding temperature and a long-term ageing process. Moreover, there exists a certain correlation between the black pixel ratio and mass loss ratio, and their relationships were demonstrated by the experimental results in this study. It is recommended that further research concentrates on the influencing mechanism and the treatment strategy for the adverse effect caused by the water used during the compaction process. The use of more types of asphalt binders, aggregate and methodologies is also recommended in further studies.

## 1. Introduction

In the past few decades, asphalt pavement has been widely used as an important type of pavement structure due to its unique advantages [1]. It is notable that asphalt binder is a common temperature-sensitive viscoelastic material and acts as a type of glue to combine the aggregate in the asphalt mixture. During the service period, the coupling effects of repeated traffic loads and environmental factors (e.g., solar radiation, moisture and temperature) result in the degradation of pavement performance and durability of the asphalt mixture, along with inducing various types of damage [2,3,4,5]. Considering the actual service conditions, a series of test specifications and performance indexes have been developed to evaluate the pavement performance of asphalt mixtures, aiming to ensure the durability and traffic safety of asphalt pavement [6,7,8,9].

Traffic safety is one of the most crucial concerns for transportation professionals. Since pavement skid resistance is of high importance to road and traffic safety, many scholars have conducted in-depth research on the influencing factors and deterioration behavior of pavement skid resistance [10]. Generally, friction coefficients and road surface texture are used to evaluate the pavement skid resistance. Previous studies have validated that pavement skid resistance is largely affected by the pavement surface texture, road–tire contact characteristics, vehicle operation parameters and environmental conditions [11,12,13]. Over the pavement’s lifetime, repeated vehicle loading would lead the pavement surface texture to be smoother and consequently deteriorates the pavement skid resistance [14]. Moreover, due to the thermosensitive property of asphalt binders, road–tire contact conditions change with the season, resulting in seasonal variations in pavement skid resistance [15]. Therefore, the investigation of pavement skid resistance is undoubtedly a complex issue due to the diversity of influencing factors.

From the road material point of view, asphalt binder removal and aggregate polishing are the main factors that affect the pavement surface texture structure and lead to pavement skid resistance deterioration [16]. Related studies have testified that important parameters such as the adhesion of asphalt binders, aggregate characteristics and aggregate gradation are crucial for pavement skid resistance [17,18,19]. There is no doubt that reasonable material selection and aggregate gradation design are the prerequisites to ensure pavement skid resistance for road administrators and contractors. Currently, researchers all over the world are focusing on the effects of material characteristics and aggregate gradation on pavement skid resistance. And the findings of published studies are considerably meaningful for pavement engineering.

However, for the newly constructed HMA pavement in China, asphalt binder removal and aggregate exposed to the environment soon after compaction, despite the use of excellent materials, may result in early deterioration [20]. Once the new pavement is open to traffic, it will accelerate the aggregate polishing and smoothing of the texture structure, which might induce premature deterioration of pavement skid resistance. In order to guarantee traffic safety, there is a need to demonstrate the cause of asphalt binder removal and aggregate exposure during the compaction process and figure out its further effect on the pavement skid resistance during the service period. On this basis, it is also of high importance to propose a strategy for mitigating the surface attenuation of HMA pavement that occurred in the early service period. However, few studies have been found that focus on the above-mentioned issue.

Besides the material properties, the compaction process also has a significant effect on the mechanical performance and durability of HMA pavement. Inadequate compaction increases the actual air void of the asphalt mixture, and hence induces various types of pavement damage [21]. Generally, air void and compactness are important indexes used to control the compaction quality. Sufficient compaction is required and some critical parameters, like rolling numbers and amplitude, are required in the Chinese specification JTG F40 2004 [7]. Unfortunately, asphalt binder removal constantly occurred during the pavement engineering, despite the adherence of the material properties and the compaction management to the corresponding specification standards. Therefore, it might be interesting to clarify the reason for the asphalt binder removal during the compaction process and after the pavement is open to traffic.

In China, it is common to spray cold water on the wheel of a road roller to prevent the hot asphalt mixture from sticking to the wheel during the compaction process of HMA pavement. Considering the similarity with the production procedure of foamed asphalt, the cold water in contact with the hot asphalt binder might turn to steam and become trapped in tiny asphalt binder bubbles. For the foamed asphalt technique, improper foamability would decrease the pavement performance of the asphalt mixture [22,23,24]. If an unexpected foaming process occurs during the compaction process of HMA pavement, the adhesive property of the asphalt binder is negatively impacted. It is well known that poor adhesion results in the degradation of the bonding strength between the asphalt binder and aggregate [25]. In this case, it increases the risk of asphalt binder removal and accelerate aggregate polishing, leading to pavement skid resistance deterioration. Moreover, asphalt binder removal could also induce raveling and moisture damage of the HMA pavement. From this point of view, it is important to demonstrate the effect of water on the surface polishing characteristics of HMA pavement during the compaction process. On this basis, it can provide guidance for mitigating the surface attenuation of HMA pavement that occurred in the early service period.

This paper aims to demonstrate whether water during the compaction period affects the surface performance of HMA pavement. Firstly, the HMA samples were prepared with different compaction temperature levels and water consumption levels. In addition, the standard long-term ageing process was also taken into consideration. Then, the modified wet-wheel abrasion test was used on the samples to simulate the traffic loading effect on the HMA pavement. Finally, the image processing method and mass loss ratio were used to evaluate the effect of water on the surface polishing characteristics of HMA pavement during the compaction process.

## 2. Materials and Methods

### 2.1. Materials

**Figure 1:**
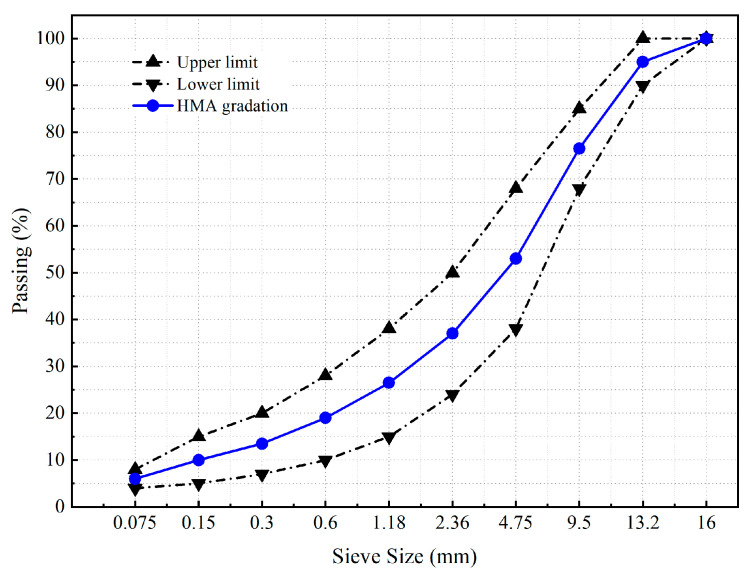
Aggregate gradation.

In this study, the AH-70 virgin asphalt binder (Wuhan, China), crushed limestone aggregate and limestone filler (Dawu, China) were used to prepare the asphalt mixture. The physical properties of these common raw materials were tested according to the Chinese standard specification JTG F40–2004. The performance indexes of the asphalt binder, aggregate and mineral filler met the technical specification requirement, and their physical properties are shown in Table 1, Table 2, Table 3, Table 4. An asphalt mixture with the maximum nominal size of 13 mm was used and the aggregate gradation is shown in Figure 1. The optimal asphalt content for the asphalt mixture was determined as 4.76% by the Marshall design procedure.

### 2.2. Sample Preparation and Test Procedure

Superpave Gyratory Compactor (SGC) samples with a diameter of 150 mm and a height of 50 mm were prepared to investigate the effect of water on the polishing characteristics of HMA. Firstly, the samples were prepared within three compaction temperature levels (120 °C, 130 °C, 140 °C) and five water consumption levels (0 L/m^2^, 0.2 L/m^2^, 0.4 L/m^2^, 0.6 L/m^2^, 0.8 L/m^2^). Considering the ageing effect, another group of samples was treated using the standard long-term ageing process, in which the loose mixture was kept in an oven at 135 °C for 4 h and the SGC samples were kept in an oven at 85 °C for 5 days. Three parallel samples were employed for each treatment parameter and the average value was obtained for the analysis. Afterwards, the modified indoor wet-wheel abrasion test was carried out on all the samples. Compared with the conventional abrasion test, the testing temperature here was set as 56 °C and controlled by a water circulating pump. Considering the actual vehicle loading, the loading level was set as 0.7 MPa. Finally, the image processing method and mass loss ratio were used for the following analysis.

### 2.3. Wet-Wheel Abrasion Test

As mentioned above, the modified wet-wheel abrasion test was used to simulate the traffic loading effect on HMA pavement in this study. Firstly, the HMA sample was put in a water bath of 56 °C for 1 h. A temperature controller and circulation pump were used to maintain the temperature of the water bath. Then, repeated loading was performed on the sample 1000 times, 2000 times, 3000 times and 4000 times, respectively. After 1000 times of the wet-wheel abrasion test, the surface image and mass value of HMA samples were obtained using the methodology in Section 2.4 and Section 2.5 for our follow-up study.

### 2.4. Image Processing Method

**Figure 2:**
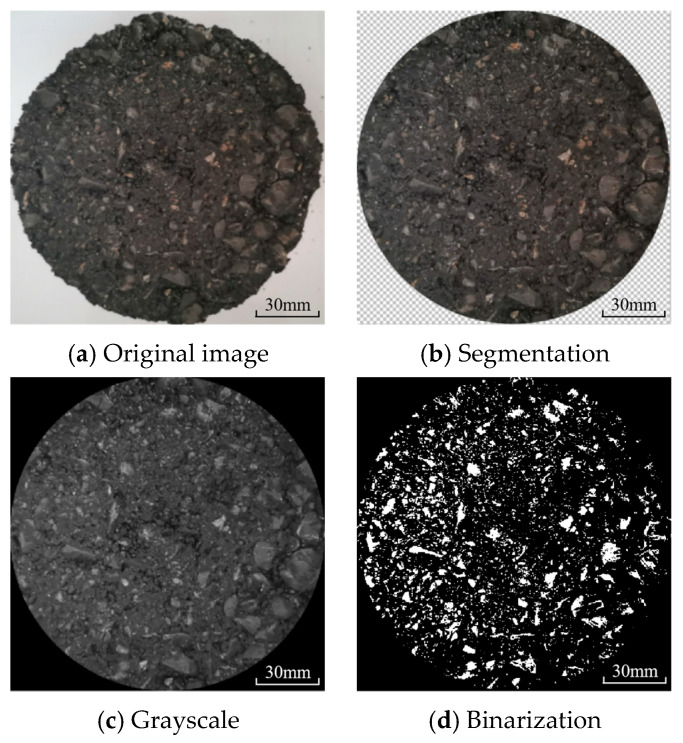
Image processing diagram.

Figure 2 illustrates the image processing diagram in this study. Firstly, the Canon PowerShot SX720 HS digital camera was used to record the surface pictures of the HMA samples. During the shooting process, the camera was placed 50 cm from the top surface of the samples each time. Then, the main wear area was obtained from the original image of the HMA sample that underwent the wet-wheel abrasion test. Afterwards, the extracted image was grayed and binarized using the MATLAB 2020 b software. Finally, the percentage of the black pixel compared to the whole area was calculated to evaluate the polishing level of the HMA sample using Equation (1). Here, the pixel size of the binarized image was 0.5 mm. If surface polishing occurred, the black pixel ratio decreased, i.e., a lower black pixel ratio means more extensive surface polishing. Therefore, the black pixel ratio was used to evaluate the surface polishing level of the HMA samples in this study.

(1) $$\mathrm{Black}\;\mathrm{pixel}\;\mathrm{ratio}=\left(1-\frac{\mathrm{Amount}\;\mathrm{of}\;\mathrm{the}\;\mathrm{Black}\;\mathrm{pixel}}{\mathrm{Amount}\;\mathrm{of}\;\mathrm{the}\;\mathrm{total}\;\mathrm{pixel}}\right)\times 100\%$$

### 2.5. Mass Loss Ratio

Due to the wearing effect, repeated loading decreased the amount of asphalt binder and aggregate in the pavement. The weight of the HMA sample decreased if surface polishing occurred. Therefore, the mass loss ratio was adopted to evaluate the surface polishing characteristics of the HMA mixture as well. The mass loss ratio can be calculated using Equation (2).

(2) $$\mathrm{Mass}\;\mathrm{loss}\;\mathrm{ratio}=\left(1-\frac{\mathrm{Mass}\;\mathrm{of}\;\mathrm{HMA}\;\mathrm{sample}\;\mathrm{after}\;\mathrm{the}\;\mathrm{abrasion}\;\mathrm{test}}{\mathrm{Mass}\;\mathrm{of}\;\mathrm{HMA}\;\mathrm{sample}\;\mathrm{before}\;\mathrm{the}\;\mathrm{abrasion}\;\mathrm{test}}\right)\times 100\%$$

## 3. Results and Discussion

### 3.1. The Effect of Water on the Surface Polishing Characteristics of HMA

#### 3.1.1. Water Consumption

**Figure 3:**
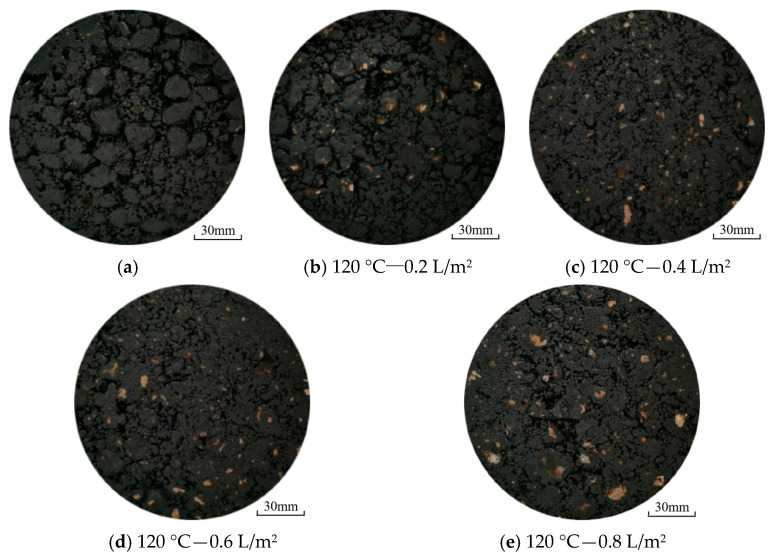
Surface images of the SGC samples molded at 120 °C with different levels of water consumption.

In this section, the effect of water consumed during the compaction process on the surface image feature of HMA samples was investigated. Figure 3 presents the surface images of the HMA samples molded at 120 °C with different levels of water consumption. For the sample prepared without water, the surface shows no evidence of polishing after the compaction process was completed. However, the surface polishing of the HMA samples was highly evident when water was used during the compaction process. The results demonstrate that the water used during the compaction process definitively induced surface polishing.

**Figure 4:**
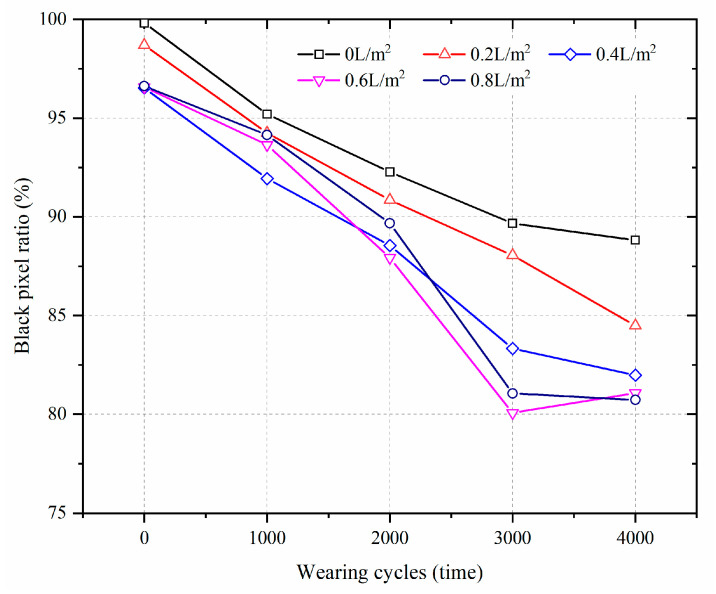
The effect of water consumption on the black pixel ratio of HMA samples molded at 120 °C during the wet-wheel wearing test.

To quantitatively analyze the change in surface image, the black pixel ratio was adopted to evaluate the effect of water consumption on the surface polishing of the HMA samples. Figure 4 illustrates the black pixel ratio of the HMA samples molded at 120 °C with different levels of water consumption and wet-wheel wearing cycles. Higher black pixel ratios mean that less polishing occurred for the HMA samples. Before the wet-wheel wearing test, the black pixel ratios of the HMA samples, which are shown in Figure 3, were 99.8%, 98.7%, 96.5%, 96.6% and 96.6% for the water consumption levels 0 L/m^2^, 0.2 L/m^2^, 0.4 L/m^2^, 0.6 L/m^2^ and 0.8 L/m^2^, respectively. It seems that surface polishing decreased when the water consumption increased from 0 L/m^2^ to 0.4 L/m^2^, while the results were almost unchanged when the water consumption increased from 0.4 L/m^2^ to 0.8 L/m^2^. For the same molding temperature, the heat of HMA dropped more rapidly when paired with greater water consumption [26]. And a lower temperature could lead to insufficient compaction and cause premature failure of the asphalt pavement, which involves surface polishing and particle stripping [27,28].

However, the effect of water consumption on the surface polishing characteristic became significant during the loading wearing cycles. Regardless of the water consumption level, the black pixel ratio of the HMA samples decreased during the wearing cycles, suggesting that the wet-wheel wearing process can result in surface polishing of the HMA samples. For the same wearing cycles, the black pixel ratio of the HMA samples decreased when the water consumption increased. It also demonstrated that the water used during the compaction process accelerated the surface polishing of the HMA samples combined with the loading effect.

#### 3.1.2. Molding Temperature

**Figure 5:**
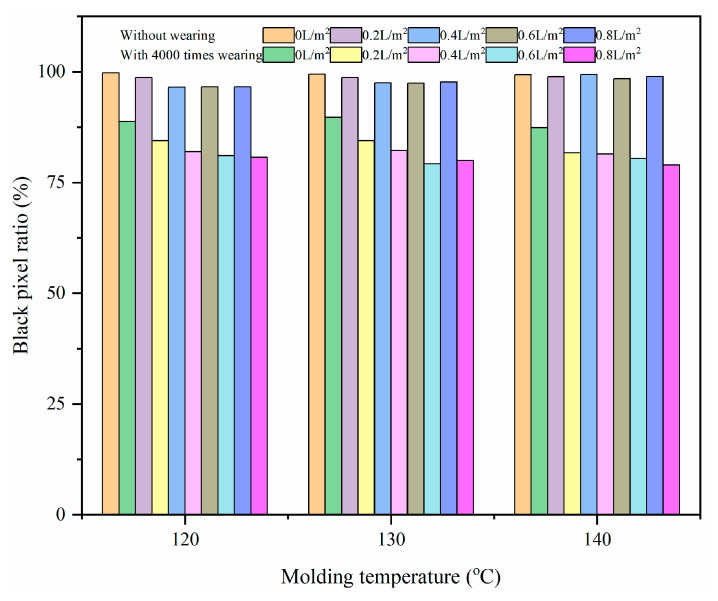
The effect of molding temperature on the black pixel ratio of the HMA samples.

To clarify the effect of the molding temperature, the samples were prepared at three temperature levels (120 °C, 130 °C and 140 °C) in this study. Figure 5 presents the black pixel ratio of the HMA samples prepared using different molding temperatures and different levels of water consumption before and after the wet-wheel wearing test. When the water consumption was 0 L/m^2^, the black pixel ratios for the HMA samples molded at 120 °C, 130 °C and 140 °C were 99.8%, 99.5% and 99.4% before the wet-wheel wearing test, and the corresponding results after the wearing test performed 4000 times were 98.7%, 98.7% and 98.9%, respectively. It implies that the molding temperature had little effect on the surface polishing performance of the HMA samples when there was no water used during the compaction process. However, the surface polishing of the HMA samples became obvious with the increasing molding temperature once water was used during the compaction process. When the water consumption was 0.4 L/m^2^, the black pixel ratios before and after the wet-wheel wearing test decreased by 7.8% and 11.6% for the HMA samples molded at 120 °C and 140 °C, respectively. It indicated that water increases the risk of surface polishing of HMA samples at a relatively high molding temperature.

For the same molding temperature, the black pixel ratio of the HMA samples decreased with the increasing water consumption, which was in accordance with the results in Figure 3. When the molding temperature was 140 °C, the decrease in the black pixel ratio before and after the wet-wheel wearing test was 1.0% for the HMA samples with 0.2 L/m^2^ water consumption. Since the water consumption increased from 0.2 L/m^2^ to 0.8 L/m^2^, the corresponding decrease in the black pixel ratio raised to 19.0%. It can be concluded that greater water consumption showed a more significant effect on the surface polishing performance of the HMA samples compared to the effect recorded when the wearing test was carried out 4000 times for a constant molding temperature.

The effect of water on the moisture resistance of asphalt mixtures is very complex. Omar et al. reviewed the theories and mechanisms related to the moisture damage of HMA. They pointed out that water is one of most common contributing factors to moisture damage [29]. On the one hand, water increases the heat loss of HMA during the compaction process. On the other hand, it also increases the risk of improper physical–chemical reactions between the cold water and hot asphalt binder. The combined effects meant that the black pixel ratio of the HMA samples showed a decreasing tend with the water consumption and compaction temperature.

#### 3.1.3. Long-Term Aging

**Figure 6:**
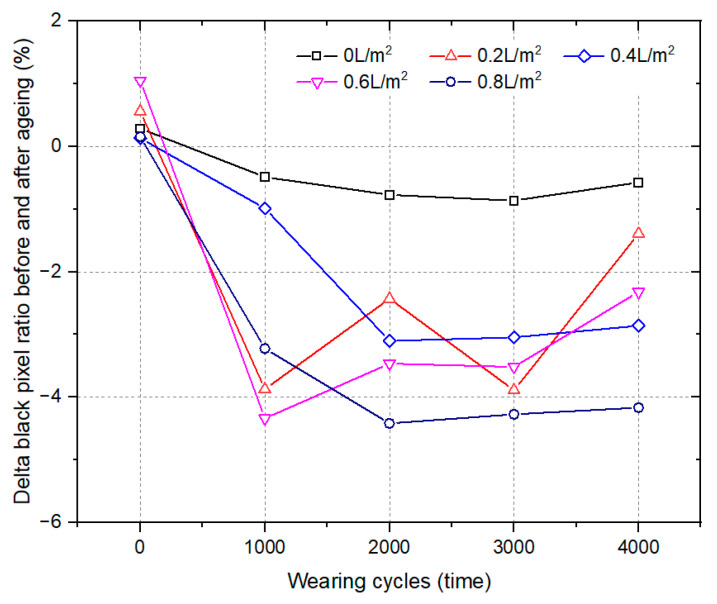
The effect of long-term ageing on the black pixel ratio of HMA samples molded at 140 °C.

Figure 6 presents the effect of long-term ageing on the surface polishing performance of the HMA samples at different levels of water consumption at 140 °C. The delta black pixel ratio was the black pixel ratio of the HMA sample after the long-term ageing process minus the corresponding result of the unaged samples. The negative value of the delta black pixel ratio indicates that the surface polishing of HMA samples become worse after long-term ageing compared to the unaged samples, while the positive value shows the opposite.

As shown in Figure 6, the delta black pixel ratio remained negative when water was used during the compaction process of the HMA samples. It was also shown that the long-term ageing process accelerates the surface polishing of the HMA samples caused by both the wearing effect and water used during the compaction process. The reason for this might be that the bonding strength of the asphalt binder weakens after ageing and stripping occurs more easily. However, the effect of water consumption on the delta black pixel ratio was not very clear after 1000 wearing cycles.

After 4000 cycles of wearing, the delta black pixel ratio was −0.6% for the sample prepared without water used during the compaction process. And the corresponding results for the samples prepared with water consumption that ranged from 0.2 L/m^2^ to 0.8 L/m^2^ were −1.4%, −2.9%, −2.3% and −4.2%, respectively. This implies that water could enhance the adverse effect of long-term ageing on the surface polishing performance of HMA samples. For the same water consumption, the delta black pixel ratio of the HMA samples decreased rapidly during the initial 2000 cycles of the wearing period and then changed slightly. It can be concluded that the water used during the compaction process had a more significant effect on the HMA sample in the initial period of loading.

### 3.2. The Effect of Water on Surface Mass Loss of HMA

#### 3.2.1. Water Consumption

**Figure 7:**
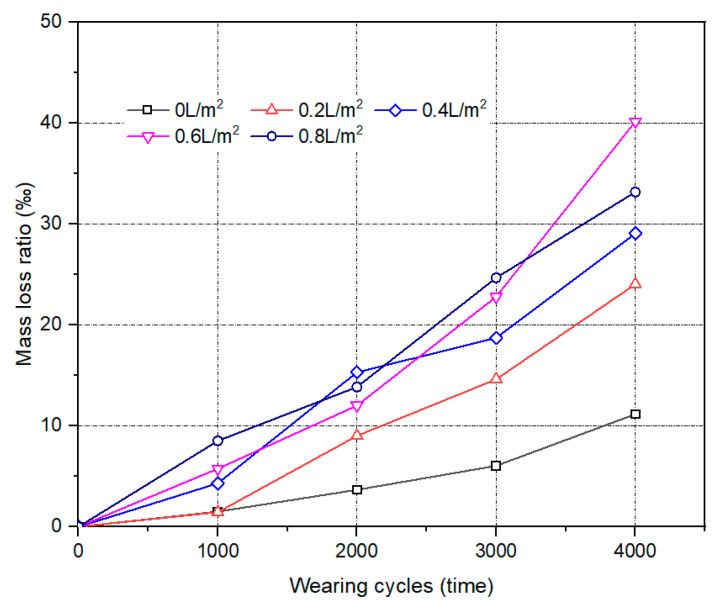
The Effect of water consumption on the mass loss ratio of HMA samples molded at 120 °C.

When surface polishing occurs, the weight of the HMA sample also decreased. From this point of view, the mass loss ratio was adopted to evaluate the effect of the water used during the compaction process on the surface polishing performance of HMA. Figure 7 presents the effect of water consumption on the mass loss ratio of HMA samples molded at 120 °C. A higher mass loss ratio means that more surface polishing occurred. Regardless of the water consumption, the mass loss ratio tended to increase with the wearing cycles. The reason for this is that the wearing effect negatively impacted the amount of asphalt binder and filler in the samples and thus decreased the mass of the samples.

For the same wearing cycles, the mass loss ratio of the HMA samples increased with increasing water consumption during the compaction process. After 3000 cycles of wearing, the mass loss ratios were 6.0‰, 14.6‰, 18.7‰, 22.8‰ and 24.7‰ for HMA samples prepared with water consumption that ranged from 0 L/m^2^ to 0.8 L/m^2^. Under the same molding temperature, the higher the water consumption, the greater the mass loss ratio of the HMA sample. It also indicated that the use of water during the compaction process would deteriorate the surface polishing performance of HMA samples, which was in accordance with the results in Figure 4.

#### 3.2.2. Molding Temperature

**Figure 8:**
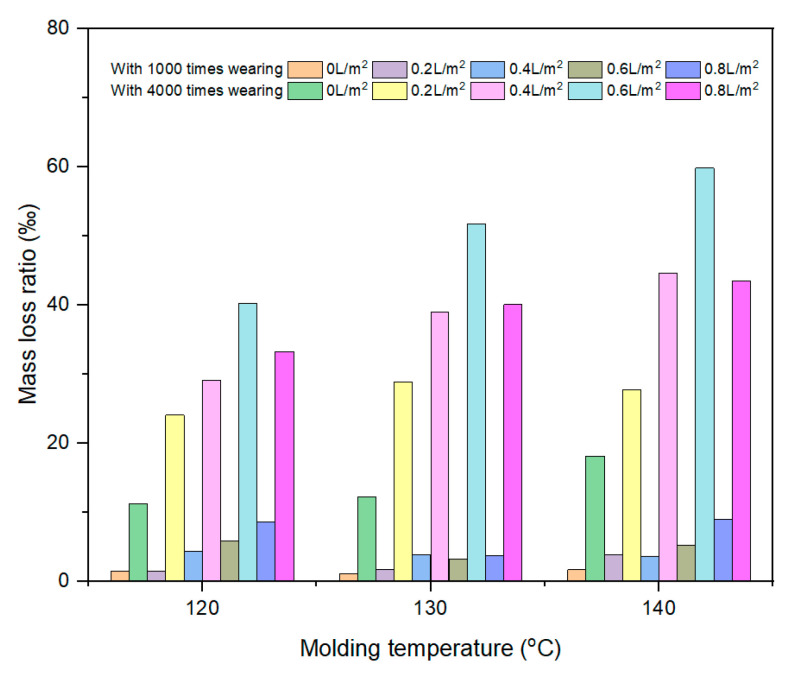
The effect of molding temperature on the mass loss ratio of the HMA samples.

Figure 8 illustrates the effect of molding temperature on the mass loss ratio of HMA samples at different levels of water consumption. For the same wearing cycles, the mass loss ratio of the HMA samples increased with increasing water consumption and molding temperature. When no water was used during the compaction process, the mass loss ratios of the HMA samples after 1000 wearing cycles were 1.5% and 1.6% at molding temperatures of 120 °C and 140 °C. When the wearing cycles increased from 1000 times to 4000 times, the corresponding results increased to 11.2% and 18.1%, respectively. It indicated that the HMA samples molded at a relatively higher temperature were more prone to surface polishing, leading to a greater mass loss during the wet-wheel wearing test.

When water was used during the compaction process, the mass loss ratio of the HMA samples was relatively greater than that without water. After 4000 cycles of wearing, the mass loss ratios for the samples prepared with 0.4 L/m^2^ water and molded at 120 °C, 130 °C and 140 °C were 29.1‰, 38.9‰ and 44.5‰, respectively. It also indicates that with the use of water during the compaction process, the HMA samples are more vulnerable to mass loss. In addition, when the water consumption was 0.6 L/m^2^, the corresponding results increased to 40.2‰, 51.7‰ and 59.7‰, respectively. It can be concluded that greater water consumption showed a more detrimental effect on the surface polishing performance of HMA samples for the same molding temperature.

#### 3.2.3. Long-Term Aging

**Figure 9:**
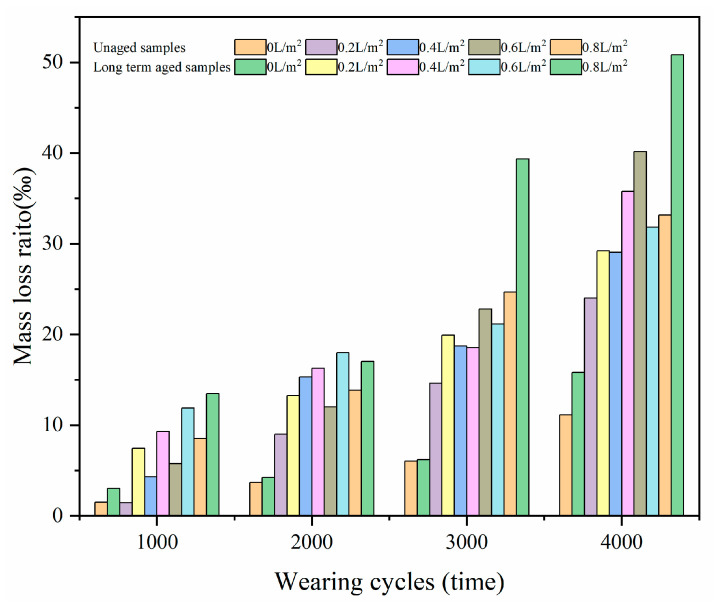
The effect of long-term ageing on the mass loss ratio of HMA samples molded at 120 °C.

Figure 9 presents the effect of long-term ageing on the mass loss ratio of HMA samples at different levels of water consumption at 120 °C. For the HMA samples prepared within the same level of water consumption and the same molding temperature, the mass loss ratio of the long-term aged samples was relatively greater than the unaged sample for the same wearing period. When there was no water used during the compaction process, the mass loss ratios of unaged and long-term aged HMA samples were 11.2‰ and 15.8‰ after 4000 cycles of wearing. When the water consumption was 0.8 L/m^2^, the corresponding results increased to 33.2‰ and 50.8‰, respectively. It also implied that the use of water during the compaction process had an adverse effect on the surface polishing performance of HMA samples and become more significant after long-term ageing.

### 3.3. Correlation Analysis of Black Pixel Ratio and Mass Loss Ratio

**Figure 10:**
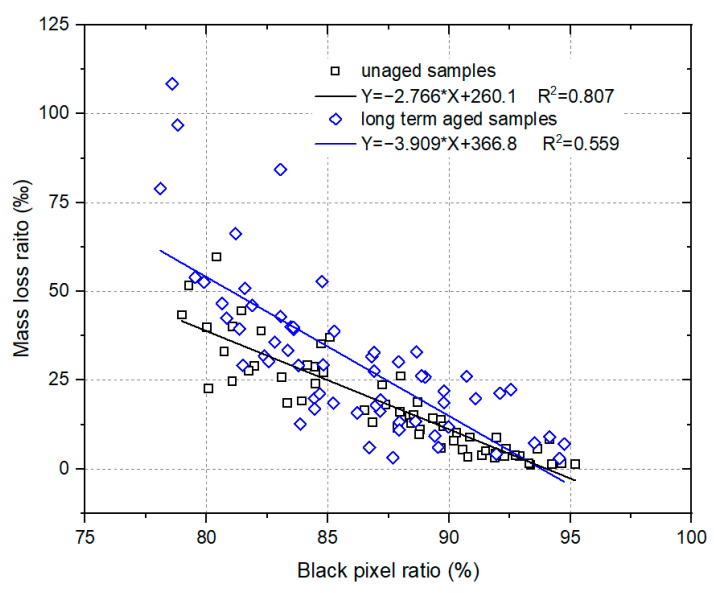
Correlation analysis of black pixel ratio and mass loss ratio for the tested HMA samples.

To better understand the effect of surface polishing, the relationship between the black pixel ratio and mass loss ratio for the tested HMA samples was analyzed and the results are shown in Figure 10. A greater black pixel ratio means that less surface polishing occurred during the wet-wheel wearing test and a lower mass loss ratio would be obtained. Therefore, the mass loss ratio decreased with the increase in the black pixel ratio for both the unaged samples and the long-term aged samples.

It was interesting that the black pixel ratio and mass loss ratio obtained in this study for the HMA samples that were prepared and tested under the same conditions, including water consumption, molding temperature, long-term ageing and wearing cycles, could be fitted by a linear equation, which is shown in Figure 10. The correlation coefficients were 0.807 and 0.559 for the unaged samples and long-term aged samples, respectively. This indicates the existence of a relationship between the black pixel ratio and mass loss ratio. Since the black pixel ratio and mass loss ratio can effectively reveal the surface polishing performance of the HMA samples, the validity of the test methods and test results can be concluded. It demonstrated that the use of water during the compaction process results in surface polishing of the HMA pavement. Therefore, further studies are needed to understand how we can eliminate the adverse effect caused by the water used during the compaction process.

## 4. Conclusions

In this study, the black pixel ratio and mass loss ratio of HMA samples were used to demonstrate the effect of water on the surface performance of asphalt pavement, considering the water consumption during the compaction process, molding temperature of specimens and the long-term ageing process. The key findings and recommendations drawn from the study are as follows:

(1) The use of water during the compaction process increases the risk of surface polishing of HMA pavement.

(2) The adverse effect of water used during the compaction process on the surface polishing characteristics became more significant for the HMA samples molded at a relatively high molding temperature and that underwent a long-term ageing process.

(3) The black pixel ratio and mass loss ratio can be used to evaluate the change in the surface polishing characteristics of HMA samples affected by the water consumption during the compaction process. There is a linear relationship between the black pixel ratio and mass loss ratio.

Overall, this study provides a reference for demonstrating the effect of water used during the compaction process on the surface polishing performance of HMA pavement. However, there are still some problems that can be explored in the future research, including the following aspects:

(1) Further research should be concentrated on the influencing mechanism of water on the surface polishing performance of the HMA mixture, including the physical–chemical reaction between the cold water and hot asphalt and how the water affects the bonding strength between the asphalt binder and aggregate.

(2) The asphalt binders and RAP materials used in this study were limited. More types of aggregate, asphalt binders and methodologies could be used to obtain a more comprehensive understanding of the adverse effect caused by the water used during the compaction process.

(3) In order to ensure the durability of HMA pavement, the proposal of a strategy to eliminate the aforementioned adverse effects in further studies is strongly recommended.

## Figures and Tables

**Table 1:** Physical properties of the AH-70 virgin asphalt binder.

Indexes	Unit	Specification Requirements	Testing Results
Penetration (25 °C, 100 g, 5 s)	0.1 mm	60–80	71
Softening point	°C	>46	48.5
Ductility (15 °C)	cm	>100	117
Kinetic viscosity (60 °C)	Pa·s	≥180	196

**Table 2:** Physical properties of the coarse aggregate.

Indexes	Unit	Specification Requirements	Testing Results
Bulk S.G.	N/A	N/A	2.715
Apparent S.G.	N/A	≥2.6	2.846
Water absorption	%	≤2	1.15
Crushing value	%	≤26	13.1
Los Angeles abrasion	%	≤28	117

**Table 3:** Physical properties of the fine aggregate.

Indexes	Unit	Specification Requirements	Testing Results
Apparent S.G.	N/A	≥2.5	2.672
Mud content	%	≤3.0	2.5
Sand equivalent	%	≥60	68
Angularity	s	≥30	39

**Table 4:** Physical properties of the mineral filler.

Indexes		Unit	Specification Requirements	Testing Results
Apparent S.G.		N/A	≥2.5	2.795
Passing percentage	0.6 mm	%	100	100
	0.15 mm		90–100	90.4
	0.075 mm		75–100	78.6
Hydrophilic coefficient		N/A	<1	0.74
Plasticity index		%	<4	3.4

## Data Availability

All the data in the tests of this study have been listed in the paper.
